# Positive Impact of Levothyroxine Treatment on Pregnancy Outcome in Euthyroid Women with Thyroid Autoimmunity Affected by Recurrent Miscarriage

**DOI:** 10.3390/jcm10102105

**Published:** 2021-05-13

**Authors:** Alessandro Dal Lago, Francesco Galanti, Donatella Miriello, Antonella Marcoccia, Micol Massimiani, Luisa Campagnolo, Costanzo Moretti, Rocco Rago

**Affiliations:** 1 Physiopathology of Reproduction and Andrology Unit, Department of Gender, Parenting, Child and Adolescent Medicine, Sandro Pertini Hospital, Via dei Monti Tiburtini 385/389, 00157 Rome, Italy; donatella.miriello@aslroma2.it (D.M.); rocco.rago@aslroma2.it (R.R.); 2 Department of Obstetrics and Gynecology, University of Rome Tor Vergata, Via Montpellier 1, 00133 Rome, Italy; francescogalanti@hotmail.it; 3 Vascular Disease and Immunology Unit, Department of Medical Area, Sandro Pertini Hospital, Via dei Monti Tiburtini 385/389, 00157 Rome, Italy; antonella.marcoccia@aslroma2.it; 4 Department of Biomedicine and Prevention, University of Rome Tor Vergata, Via Montpellier 1, 00133 Rome, Italy; micol.massimiani@unicamillus.org; 5 Saint Camillus International University of Health Sciences, Via di Sant’Alessandro, 8, 00131 Rome, Italy; 6 Department of Systems’ Medicine, University of Rome Tor Vergata, UOC of Endocrinology and Diabetes, Section of Reproductive Endocrinology Fatebenefratelli Hospital, ‘Isola Tiberina’, Via di Ponte Quattro capi, 39, 00186 Rome, Italy; moretti@med.uniroma2.it

**Keywords:** levothyroxine, recurrent miscarriage, pregnancy, euthyroid women, thyroid autoimmunity

## Abstract

Impaired thyroid hormone availability during early pregnancy is associated with recurrent miscarriage (RM) and adverse pregnancy outcomes. The main cause of thyroid dysfunction is thyroid-related autoimmunity (TAI), characterized by a significantly higher serum level of thyroid-stimulating hormone (TSH) compared to that of women without thyroid autoimmunity. TAI is associated with a significantly increased risk of miscarriage, and the incidence of TAI in women experiencing RM is higher compared to normal fertile women. In the present study, we have performed a retrospective analysis comparing the ability to conceive, the number of miscarriages and full-term pregnancies between 227 euthyroid women with autoimmune thyroid disease affected by RM and treated with levothyroxine (LT4) as adjuvant therapy, and a control group of 230 untreated women. We have observed a significant improvement of full-term pregnancies in treated women (59%) compared to untreated women (13%, *p* < 0.0001). Compared to the control group, treated women had a lower percentage of miscarriages (12% vs. 30%) and improved capacity to conceive (57% vs. 29%). Using age as a variable, the outcome in women younger than 35 years was not influenced by the LT4 therapy. Whereas, in women over 35 years, supplementation with LT4 significantly reduced the miscarriage rate (*p* < 0.05). We can conclude that a transient impairment of TH availability, not easily detectable before pregnancy, could be an important cause of RM in a subset of euthyroid women with autoimmune thyroid disease. This transient impairment may be reverted using adjuvant treatment with low doses of LT4.

## 1. Introduction

During normal gestation, thyroid hormone (TH) availability increases in order to meet the physiological demand of the growing fetal placental unit [1]. The prevalence of hypothyroidism during pregnancy is estimated to be 0.3–0.5% for overt hypothyroidism (OH) and 2–3% for subclinical hypothyroidism (SH) [2]. Thyroid autoantibodies are found in 5–18% of women in childbearing age, and chronic autoimmune thyroiditis (TAI) is the main cause of hypothyroidism during pregnancy. Thyroid dysfunction is associated with recurrent miscarriages, and the risk of early abortion is substantially raised with the presence of thyroid autoimmunity, even if there was a condition of euthyroidism before pregnancy [3,4]. The association between TAI and miscarriage remains a matter of debate: it has been suggested that women with TAI have a more generalized autoimmune activity [5,6], leading to an increased fetal loss. Recent data suggest that euthyroid women are affected by TAI-impaired embryo implantation through induction of endometrial receptivity defects [7]. However, few studies have demonstrated the efficacy of therapies which modulate the immune function in preventing miscarriage [8]. It is plausible that, in women affected by TAI, the thyroid gland may have an inadequate thyroid hormones release during early pregnancy. The increasing of miscarriage rate in women affected by TAI could be due to a thyroid dysfunction, rather than a generalized over-reaction of the immune system [9]. Furthermore, a local action of THs on the female reproductive organs and the embryo during implantation has to must be considered crucial for a successful pregnancy, as well [10]. Therefore, TSH may be a factor influencing ovarian reserves, and elevated TSH levels may have a deleterious effect on ovarian function [11,12]. In women with treated primary hypothyroidism, it has been demonstrated that an increase in pre-pregnancy doses of Levothyroxine (LT4), up to 26%, at confirmation of gestation or when seeking pregnancy guarantees an adequate hormonal level in early pregnancy. Moreover, a pre-implantation level of TSH around 0.5 mIU/L is protective against TSH elevation over 2.5 mIU/L in the first trimester [13]. Although it has been described as a significant increase in the rate of pregnancy loss in thyroid antibody-negative women with a TSH level between 2.5 and 5 mIU/L [14,15,16], the effect of LT4 treatment in euthyroid women has been evaluated only in the presence of AITD, which however demonstrates that LT4 is able to reduce the number of miscarriages and premature deliveries [17,18]. Despite this fact, a recent Cochrane meta-analysis [19], including non-randomized controled trials, shows conflicting data concerning levothyroxine treatment in subclinical hypothyroidism or in euthyroid women with AITD.

In our retrospective study, we have evaluated the ability of low-dose levothyroxine treatment to prevent miscarriages in a group of apparently euthyroid women with TAI and a history of unexplained recurrent miscarriage. Although in early pregnancy the normal range of serum TSH concentration has been established between 0.4 and 2.5 mIU/mL [18], and treatment is encouraged only for women planning a pregnancy if the serum TSH values are between 2.5 and 4 mIU/mL, we have treated all women affected by recurrent miscarriage, independently from the TSH baseline value, in order to explore the effect that a tailored dose of LT4 may improve the outcome in pregnancy.

## 2. Materials and Methods 

### 2.1. Patients

Our retrospective study includes 557 non-pregnant Caucasian women affected by autoimmune thyroid disease and with a history of unexplained, recurrent miscarriages. All patients attended the Day Hospital for Recurrent Miscarriage of the Fatebenefratelli Hospital “San Giovanni Calibita” and Unit of Physiopathology of Reproduction of Sandro Pertini Hospital of Rome, Italy, between 2015 and 2019. This study was conducted following the Ethical Principles of the Helsinki Declaration and national laws. The majority of women included in this study (85%) had three or more episodes of miscarriage, while a smaller group (15%) had only two episodes. In this study, we did not include 74 women affected by thyroid nodules, thyroid cancer, or those having taken levothyroxine previously and 123 women positive for antinuclear antibodies, antibodies against phospholipids and cofactors, lupus anticoagulant, smooth muscle, and mitochondria. We also excluded 70 women with other possible causes of RM, including anatomical, endocrinological, and genetic alterations, as well as uterine abnormalities, uterine myomas, and parental chromosomal abnormalities. All patients underwent hysterosonosalpingography, and, whenever indicated, hysteroscopy and laparoscopy as part of their work-up.

We considered the potential influence of male factor and semen quality was microscopically evaluated for all partners of women involved in the study. Although DNA fragmentation was not evaluated, total sperm concentration number, morphology, and progressive motility appeared normal (not shown).

### 2.2. Thyroxine Treatment

Regarding our sample of 557 women, 100 had a pre-conceptional TSH blood level higher than 2.5 mIU/L and were treated with LT4, and not included in our study. Out of the 457 women, 227 received LT4 adjuvant therapy, while 230 did not. Control and treated women shared similar age and similar BMI. Levels of FT4 and FT3 in both groups were also evaluated. All patients’ characteristics are summarized in Table 1.

At initial screening at least three months before hCG detection (basal time or Time 0), serum levels of Follicle Stimulating Hormone (FSH), luteinizing hormone (LH), Anti-Müllerian hormone (AMH), prolactin, estradiol, progesterone, thyroid-stimulating hormone (TSH), free-Thyroxine (FT4), free triiodothyronine (FT3), TPO-Ab, and TG-Ab were assessed. In addition to routine hormonal checks, a dynamic evaluation of thyrotrope function was performed in the early follicular phase after overnight fasting by injecting i.v. 200 mcg Thyrotropin Releasing Hormone (TRH) and measuring of TSH level at 0, 20, and 40 min [20]. This was used to fine tune a therapeutic regimen of LT4 therapy based on each TRH-induced TSH rise above the mean of a healthy population.

All patients with a TRH-stimulated TSH response >15 mIU/L at 20 min were treated with LT4. All thyroid evaluations (FT3, FT4, TSH) were re-performed at the time of hCG first detection (Time 1) and at around 12 to 15 weeks of gestation (Time 2), with results considered for statistical evaluation.

The dose of LT4 was decided according to bTSH level and TRH-stimulated TSH test at time 0 (0.4 ≤ TSH ≤ 1.5 mIU/L = 39.50 mcg ± 12.46; 1.5 < TSH ≤ 2.0 mIU/L = 40.28 mcg ± 12.30; 2.0 < TSH ≤ 2.5 mIU/L = 50.00 mcg ± 9.28). Moreover, in the group with TSH > 2.5 mIU/L before pregnancy, we started the pre-conceptional treatment (54.54 mcg ± 9.75). Considering the TSH level, clinicians decided to adjust LT4 dosage for each patient and to repeated hormonal dosage when needed. No pregnancy (NP), full-term pregnancy (clinical pregnancy ≥37 weeks, FT-P), and miscarriages (M) were recorded and compared between treated and untreated patients.

### 2.3. Analytical Methods

Serum TSH, FT3, FT4, and prolactin were measured using a highly sensitive electrochemiluminescent immunoassay (Roche, Mannheim, Germany). The measurement range for TSH was 0.005–100.0 mIU/L. The intra-assay CV was 2%, and the inter-assay CV was 7.2% with an analytical sensitivity of 0.005 mIU/mL. The FT4 measurement range was 0.23–77.70 pg/mL (0.300–100.0 pmol/L). The intra-assay CV was 2%, and the inter-assay CV was 4.8%, with an analytical sensitivity of 0.300 pmol/l. The FT3 measurement range was 0.26–32.50 pg/mL (0.400–50.0 pmol/L). The intra-assay CV was 2%, and the inter-assay CV was 3.4%, with an analytical sensitivity 0.400 pmol/L. Anti-TPO and anti-Tg auto-antibody levels were also determined using the same electrochemiluminescent immunoassay system (Roche, Mannheim, Germany). TPO and hTG antibodies were considered positive when titles exceeded 65 IU/mL for TPOAb and 115 IU/mL for TgAb. Serum AMH levels were measured using the AMH GenII enzyme-linked immunosorbent assay kit (functional sensitivity: <0.16 ng/mL; Medical & Biological Laboratories Co., Ltd., Nagoya, Japan).

### 2.4. Statistical Data Analysis

A comparison of the variables between women affected with unexplained RM, and the control group, was performed by means of the Student’s *t*-test after the appropriate transformation to improve gaussianity, reducing heteroscedasticity, in order to avoid the effect of outliers. Whenever heteroscedasticity could not be reduced adequately, a correction to the degrees of freedom was applied. Data are given as mean and SD and compared by the Student’s *t*-test. Pregnancy, abortion, and delivery rates were analyzed by two-tailed Fisher’s exact test and product limit estimates with 99% confidence limits.

## 3. Results

### Basic Characteristics and Clinical Outcomes

Considering 457 women with preconceptional TSH blood level ≤2.5 mIU/L, 227 received LT4, adjusted during the follow-up, while 230 did not receive therapy.

All data and results are reported in Table 2. 

The association between treatment and full-term pregnancy resulted statistically significant: 134 women (59%) vs. 29 women (13%) (*p* < 0.0001). In particular of the treated women, 134 (59%) had a full-term pregnancy, 28 (12%) experienced a miscarriage, and 65 (29%) did not conceive; in the untreated group, 29 (13%) had a full-term pregnancy, 69 (30%) experienced a miscarriage, and 132 (58%) did not conceive. 

Dividing patients according to bTSH value, the association between treatment and full-term pregnancy was statistically significant for all TSH levels between 0.4 ≤ TSH ≤ 1.5 mIU/L, 1.5 < TSH ≤ 2.0 mIU/L and 2.0 < TSH ≤ 2.5 mIU/mL (*p* < 0.001). Therefore the both associations between treatment and miscarriage and treatment and no pregnancy resulted statistically significant for TSH levels between 0.4 ≤ TSH ≤ 1.5 mIU/L and 1.5 < TSH ≤ 2.0 mIU/L (*p* < 0.001) (Table 2). 

Regarding administered therapy, women with miscarriage needed a higher dose of LT4 with respect to women who achieved a full-term pregnancy (*p* = 0.0184). Furthermore, women who continued to abort needed a higher increase of LT4 during the follow-up with respect to women with full-term pregnancies (11.61 ± 15.93 vs. 4.66 ± 10.25; *p* = 0.0039) (Table 3).

**Figure 1:**
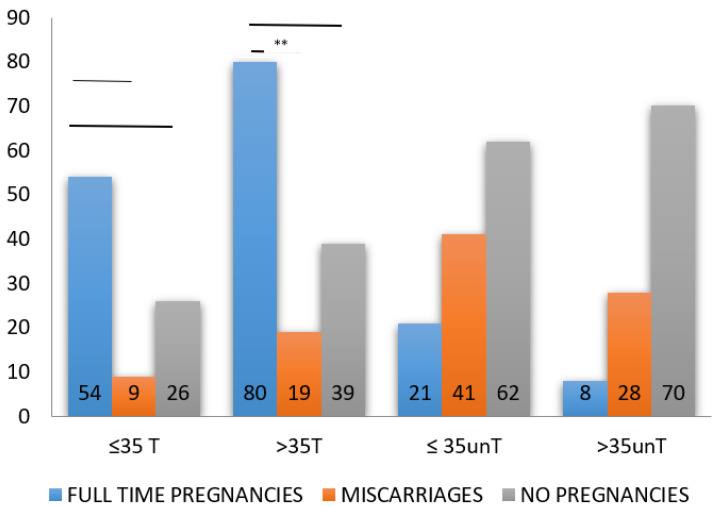
Histograms showing the outcome of women affected by recurrent miscarriage according to age. ≤35T = women up to 35 years treated with LT4. >35 = women older than 35 years treated with LT4. ≤35unT = women up to 35 years not treated with LT4. >35 unT = women older than 35 years not treated with LT4. **, statistically significant.

By dividing patients according to their age, the positive effect of LT4 treatment in the achievement of full-term pregnancies appears statistically significant for patients over 35 years of age: F-TP (80 women) vs. M (19 women) (*p* < 0.01); F-TP (80 women) vs. NP (39 women) (*p* < 0.05). LT4 treatment appears not significantly associated with positive pregnancy outcome in women under the age of 35 (Figure 1).

## 4. Discussion

This study was designed with the aim of evaluating the potential benefit of LT4 treatment in euthyroid women with TAI experiencing recurrent miscarriages. Furthermore, the increasing incidence of pregnancy loss in women with TSH higher than 2.5 mIU/L provides evidence to support the treatment of pregnant women according to this upper limit [14]. However, the timing for the increase of LT4 requirement during pregnancy is still controversial. In fact, on one hand, the switching from a replacement to a partially suppressed LT4 regimen, performed once a patient has decided to look for a pregnancy, results in adequate serum FT4 levels up to the first post-conception endocrinological consultation [21]. On the other hand, the etiology of hypothyroidism seems to play a pivotal role in determining the timing and magnitude of TH adjustment. Patients require careful monitoring of thyroid function upon confirmation of conception and anticipatory adjustments to LT4 dosing, based on the etiology of their hypothyroidism [22]. ATA guidelines [23] recommend that all treated hypothyroid women (currently receiving LT4) optimize thyroid status preconception. According to clinical trials, maternal serum TSH concentration of <2.5 mIU/L is indicated as a reasonable goal for all these women. Ideally, lower TSH values (<1.5 mIU/L) will likely further reduce the risk of mild hypothyroidism in early pregnancy. Apart from hypothyroidism and subclinical hypothyroidism developed during pregnancy, euthyroid women with TAI experience dramatical changes in their demand for TH production and biological action as early as the first trimester of pregnancy [24]. The preparation of the endometrium and the following implantation is crucial to establish pregnancy, whose maintenance of which is dependent on a multitude of systemic and local endocrinological events [15,25]. Therefore, an adequate thyroid reserve is crucial for successful implantation and pregnancy. The 20–25% of otherwise unexplained early-pregnancy losses could be due to a lack of physiological endocrine adaptation that follows implantation. The present study demonstrates that treating TAI euthyroid women, affected by RM, with LT4 can prevent a further miscarriage. A marked decrease in both miscarriage and preterm delivery has been observed in euthyroid women affected by TAI when treated with LT4 [18]. Endocrine Society Guidelines suggest treating women with subclinical hypothyroidism with LT4 during pregnancy in order to prevent obstetrical complications in both women with and without TAI [26].

The evidence of our work was that 134 out of 227 treated women succeeded to obtain a full-term pregnancy. The strength of our work was to start treating these women before pregnancy, by at least three months, offering the possibility to monitor their reaction and adaptation to the therapy, without inducing a rushing correction and stressing the patient. It is important to underline that the LT4 treatment did not induce hyperthyroidism in any patient. The safety of not performing a suppressive LT4 treatment in women who are planning pregnancy has been increasingly questioned, as high FT4 levels exert a toxic effect on the fetus, increasing the miscarriage rate [27]. The overtreatment was avoided by not treating women with TRH-stimulated TSH level lower than 15 mUI/L, detected 20 minutes after the bolus, or with an increase lower than 10 mIU/L [20], and assuring a strict evaluation of thyroid function before pregnancy.

Starting LT4 treatment before pregnancy, in women otherwise considered as euthyroid, possibly helped to avoid early thyroid impairment at the beginning of pregnancy. These women were not infertile but were affected by RM. As a consequence, their pregnancy could be saved and restored, promptly assuring a proper TH supply. Assuring an adequate level of THs during the first 10–12 weeks of pregnancy is mandatory [13], as THs might be involved in endometrium preparation to pregnancy and initial trophoblast development. Our results confirm and expand previous data reported by Rotondi and colleagues, showing that preconception adjustment of LT4 may result in adequate maternal thyroid function up to the first post-conception evaluation [21]. The preconceptional dose of LT4 prevented the TSH increase at ß-HCG detection. In our study, the overall increase of gestational LT4 dose requested by patients who succeeded to obtain full-term pregnancy was lower compared to the one reported by a previous study [28]. In this study, the initial dose the authors used in subclinical hypothyroid women, diagnosed during gestation, was 74.4 ±14.5 mcg/day. The final dose was 95.9 ± 21.7 mcg/day, with a 29% increase in the dose. In our study, patients that had a full-term pregnancy received an initial dose of 42.16 ± 12.42 mcg/day, starting before pregnancy, while the final dose was 46.83 ± 14.52 mcg/day. The timing of LT4 initial treatment may account for such difference: starting treatment with a lower LT4 dosage before pregnancy may better reproduce the hormonal physiology than starting therapy with higher doses of hormones at the beginning of pregnancy. Another relevant aspect to discuss is that Verga and colleagues [28] reported to use of a starting dose of LT4 during gestation higher than the dose administered to those already treated before pregnancy. However, at the end of pregnancy, they all reached a similar dosage of substitutive therapy. Our patients did not come to assume a mean dose of LT4 such high; as a consequence, this observation can suggest the necessity to consider a new shade in the spectrum of thyroid disease during pregnancy, apart from subclinical hypothyroidism. According to previous data, a two-tablet increase in LT4, started at confirmation of pregnancy, significantly reduces the risk of maternal hypothyroidism during the first trimester and mimics normal physiology [29].

A recent multicenter, randomized, placebo-controlled study showed that, among TAI women, the use of LT4 did not result in a higher rate of live birth compared to placebo, nor it showed a significant between-group difference in other pregnancy outcomes, including pregnancy loss [30]. These results are in contrast with our observations, however the reported randomized study included both women affected by RM and infertility, while in our study we only included women affected by RM. The stringent selection in the recruitment of our study group, which did not include women with infertility problems, could explain, at least in part, the difference between the two studies. An additional difference that could explain these contrasting results may reside in the levels of TSH of the patients included in the study. Indeed, we included only women with TSH ≤ 2.5 mIU/L and we adapted the therapy to each single patient, while Dhillon-Smith et al. [30] included both TSH ≤ 2.5 and TSH > 2.5 mIU/L; in the group of women with TSH ≥ 2.5 the fixed dose of LT4 could not be ameliorative for the obstetric outcomes.

In our study, assuming that TSH and FT4 measurements are mandatory in pregnant patients, we adjust the LT4 dose according to vigilant monitoring of thyroid function. To conclude the former, it is remarkable to underline that the evidence of the present study is an adequate treatment before pregnancy, a strict monitoring of thyroid function before and during gestation, and a tailored and prompt adjustment of levothyroxine dose according to TSH serum values. An important limitation of the present study is represented by its retrospective nature, so that our results need to be confirmed by fugure randomized controlled trial. Another potential limitation is the missed correlation between TSH level and hCG blood concentration at the time of miscarriage. Furthermore, it should be noted that there was a difference in the age of treated and non-treated patients, although slight; BMI, incidence, and miscarriage history before enrollment was similar. Our study included only women of caucasian race and the lack of ethnic heterogenicity may be an important limitation.

Another aspect concerns the trend observed in women with recurrent pregnancy loss who continued to miscarry. These patients showed a good response to therapy up to the first beta-hCG detection, after which they started to require a higher dose of LT4, according to their TSH level. An explanation to this phenomenon is probably hidden behind the biological effects mediated by the binding of THs with their receptors (TRs) in early pregnancies, effects modulated by the interactions with deiodinases [31]. The “resistance” of endometrial tissue to increased doses of THs can be searched in a defective message elicited by T3/T4 in such cells. Even though T3/T4 concentration in the blood is crucial to activate intracellular thyroid-dependent pathways, it is necessary that all membrane/nuclear receptors and signal transducers must work properly to allow THs to fulfill their role. This implies that the TH signal needs blood thyroxine vehicles, membrane and intracellular transporters, deiodinase activity, TH nuclear receptors and coactivators, and corepressors to be correctly expressed. It is evident that, in addition to the main feed-back regulation of the HPT axis, relevant mechanisms, such as deiodinase expression and TH transporters, may be critical for the biological effects mediated at a cellular level by TH during the first weeks of pregnancy. In a subset of healthy women, the disruption of these mechanisms may be responsible for transient thyroid impairment in early pregnancy. An imbalance of even one of the complex mechanisms discussed may explain the reason why some euthyroxinemic women may experience a transient impairment of thyroid function in early pregnancy. The need for an adequate supply of maternal TH in the maintenance of early pregnancy emphasize the importance of thyroid-dependent mechanisms in the organization of early placental tissue.

## 5. Conclusions

In conclusion, euthyroid women with TAI affected by RM seem to benefit from LT4 treatment. Our results confirm that the optimal timing to start the LT4 treatment is at least three months before pregnancy, with a tailored therapy and a strict follow-up. The switch from endocrinology to intracrinology is necessary when molecular mechanisms and pathways start to be described and when the hormone activity resulted to be finely regulated by epigenetics and pre/post-translational modifications encountered from blood to cellular nuclear receptors.

Finally, it is remarkable to underline that the present study proves a strict monitoring of thyroid function before and during gestation and suggests a tailored and prompt adjustment of LT4 during early pregnancy. Furthermore, it is important to remember monitoring iodine intake also in women living in iodine-sufficient areas and assuming iodine supplementation as recommended, because it could be insufficient during pregnancy. To conclude, we propose that a low doses of LT4 might avoid the disruption of molecular mechanisms activated by TH around the implantation window, which may be responsible, in a subset of healthy women, of transient thyroid impairment in the early pregnancy.

## Figures and Tables

**Table 1:** Characteristics of patients enrolled in the study: Treated patients and untreated patients had similar characteristics.

	Treated Patients	Untreated Patients	*p*-Value
	(*n* = 227)	(*n* = 230)	
Age (years)	36.30 ± 4.36	34.74 ± 4.36	0.31
BMI (kg/m^2^)	23.9 ± 2.73	23.64 ± 2.57	0.56
Previous miscarriage (number)	3.1 ± 2.01	3.3 ± 1.37	0.58
AMH ng/ml	2.1±0.9	2.5±1.3	0.63
bTSH (mIU/L)	1.72 ± 0.44	1.37 ± 0.51	0.25
TSH + 20’ (mIU/L)	15.72 ± 4.52	10.54 ± 4.87	0.04
bFT4 (pg/mL)	11.06 ± 1.65	9.34 ± 5.17	0.32
bFT3 (pg/mL)	2.94 ± 0.65	3.0 ± 1.2	0.44
Ab TPO (IU/mL)	79 ± 2.1	81 ± 1.7	0.22
Ab Tg (IU/mL)	144 ± 1.33	139 ± 0.98	0.19

Abbreviation: b = basal time. All data are represented as mean ± SD.

**Table 2:** Data collected from euthyroid polyabortive women treated with levothyroxine (LT4) according to LT4 and NO LT4 treatment and bTSH value (0.4 ≤ TSH ≤ 1.5; 1.5 < TSH ≤ 2.0; 2.0 < TSH ≤ 2.5).

	LT4 F-T Preg. (*n* = 134)	NO LT4 F-T Preg. (*n* = 29)	*p-*Value	LT4 Miscarr. (*n* = 28)	NO LT4 Miscarr. (*n* = 69)	*p-*Value	LT4 NO Preg. (*n* = 65)	NO LT4 NO Preg. (*n* = 132)	*p-*Value
Age (years)	36.21 ± 4.14	32.96 ± 4.79	(0.0003)	37.43 ± 4.69	33.99 ± 4.86	(0.0019)	36 ± 4.65	35.52 ± 3.81	NS (0.44)
bTSH (mIU/L)	1.65 ± 0.46	1.44 ± 0.61	(0.0364)	1.89 ± 0.40	1.54 ± 0.53	(0.0016)	1.78 ± 0.37	1.27± 0.43	(0.0001)
TSH + 20’(mIU/L)	15.75 ± 4.54	9.73 ± 2.92	(0.0001)	16.19 ± 4.59	12.40 ± 5.66	(0.0023)	15.34 ± 4.49	9.03 ± 3.63	(0.0001)
TSH 1 (mIU/L)	0.97 ± 0.42	1.31 ± 1.41	(0.0172)	0.89 ± 0.37	/	/	1.03 ± 0.43	/	/
TSH2 (mIU/L)	1.11 ± 0.55	1.30 ± 0.45	NS (0.08)	1.27 ± 0.60	/	/	/	/	/
0.4 ≤ TSH ≤ 1.5 mIU/mL	50	16	(<0.001)	7	32	(<0.001)	20	94	<0.001
1.5 < TSH ≤ 2.0 mIU/mL	54	7	(<0.001)	9	23	(<0.001)	24	31	<0.001
2.0 < TSH ≤ 2.5 mIU/mL	30	6	(<0.001)	12	14	NS (0.4)	21	7	NS 0.006

LT4 = treatment; NO LT4 = no treatment; F-T Preg. = full term pregnancy; Miscarr. = miscarriage; NO Preg. = no pregnancy; bTSH = basal TSH; TSH1 = correspondent to hCG detection; TSH2 = 12–15 weeks of pregnancy.

**Table 3:** Characteristic of total patients treated with LT4.

	TOT F-T Pregnancy (*n* = 134)	TOT Miscarriage (*n* = 28)	TOT No Pregnancy (*n* = 65)
LT4 *t*0 (μg/die)	42.16 ± 12.42	42.86 ± 13.36 (*p* = 0.79 NS)	42.31 ± 11.63 (*p* = 0.93NS)
LT4*t*1 (μg/die)	44.22 ± 12.61	46.43 ± 17.63 (*p* = 0.43 NS)	43.08 ± 12.11 (*p* = 0.54 NS)
LT4*t2* (μg/die)	46.83 ± 14.52	54.46 ± 19.31 (*p* = 0.0184)	/
Dose Incr1	2.05 ± 6.89	3.57 ± 13.11 (*p* = 0.37 NS)	0.77 ± 4.35 (*p* = 0.17 NS)
Dose Incr 2	2.61 ± 8.82	8.04 ± 13.70 (*p* = 0.0086)	/
Dose Incr Tot	4.66 ± 10.25	11.61 ± 15.93 (*p* = 0.0039)	/

## Data Availability

The data presented in this study are available upon request from the corresponding author. The data are not publicly available due to privacy issues.

## Abbreviations

TAI	Thyroid-related autoimmunity
RM	Recurrent miscarriage
LT4	Levothyroxine
TH	Thyroid hormone
OH	Overt hypothyroidism
SH	Subclinical hypothyroidism
FT4	Free-Thyroxine
TSH	Thyroid-stimulating hormone
T3	Triiodothyronine
TR	Thyroid hormone receptor
